# Viruses in the sediments around the trenches harbor diverse survival mechanisms

**DOI:** 10.1002/imo2.70124

**Published:** 2026-07-26

**Authors:** Yue Su, Hao Yu, Shujuan Sun, Kaiyang Zheng, Ziyue Wang, Chen Gao, Guangxue Li, Xiaoshou Liu, Hongbing Shao, David Paez‐Espino, Andrew McMinn, Yantao Liang, Min Wang

**Affiliations:** ^1^ MoE Key Laboratory of Evolution and Marine Biodiversity, College of Marine Life Sciences, Institute of Evolution and Marine Biodiversity, Frontiers Science Center for Deep Ocean, Center for Ocean Carbon Neutrality Ocean University of China Qingdao China; ^2^ Haide College Ocean University of China Qingdao China; ^3^ College of Marine Geosciences Ocean University of China Qingdao China; ^4^ UMT‐OUC Joint Academic Centre for Marine Studies Qingdao China; ^5^ Ancilia Biosciences, Inc., Alexandria Launch Labs New York New York USA; ^6^ Institute for Marine and Antarctic Studies University of Tasmania Hobart Tasmania Australia

## Abstract

Deep‐sea trenches are the deepest and most isolated environments in the global ocean but the diversity and adaptation of the viral communities remain poorly characterized. In this study, 5563 viral operational taxonomic units (vOTUs) were identified from sediments of the Yap Trench and Western Caroline Basin. More than 99% of these vOTUs could not be assigned to known viral families and showed limited similarity to sequences in current global viral databases, indicating substantial novelty. Viral community composition and diversity were found to significantly vary with water depth. The vOTUs also encoded a diverse range of functional genes possibly related to their ability to survive in extreme deep‐sea sediments, including genes involved in lipid metabolism, DNA modification, and immune evasion. The results show that deep‐sea trench sediments contain highly distinct viral communities structured by depth‐associated environmental variations.

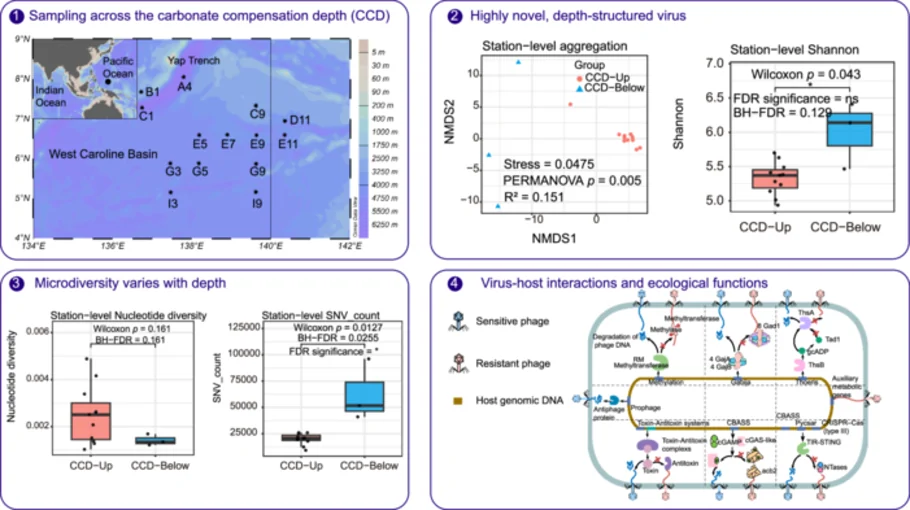

To the Editor,

Deep‐sea trenches (Hadal trenches), defined as marine habitats deeper than 6000 m below sea level, are the deepest and among the least explored environments in the ocean [1]. Extreme hydrostatic pressure, near‐freezing temperatures and focused organic carbon delivery via turbidity currents generate organic‐rich sediments that sustain intense benthic microbial activity [1]. Viruses, as the most abundant biological entities in the ocean, are likely to play important roles in these ecosystems [2]. The Oceanic Trench Viral Genome Dataset (OTVGD), constructed from samples from the Mariana, Yap, and Kermadec Trenches, highlighted the genomic novelty, dynamic behavior, and environmental diversification of viruses within hadal trenches [3]. Trench‐specific viral assemblages and auxiliary metabolic genes (AMGs) potentially involved in carbon and sulfur cycling were further identified from Mariana Trench's Challenger Deep sediments [4, 5]. The presence of these AMGs suggests that viruses may influence the hadal ecosystem through reprogramming the host's metabolism and regulating key microbial communities [4, 5]. Furthermore, the enhanced prevalence of temperate viruses and integrase genes in hadal regions is indicative of a widespread temperate life strategy [4, 5].

Host‐virus interactions are shaped by reciprocal evolutionary pressures. Bacteriophages exert strong selective pressure on prokaryotes, which have evolved both innate and adaptive immune defenses [6]. Innate immune defenses, including Restriction Modification (RM) systems and abortive infection (Abi), whereas adaptive immunity is mainly mediated by adaptive mechanisms that include the clustered regularly interspaced short palindromic repeats/CRISPR‐associated protein system (CRISPR/Cas systems) [6]. Correspondingly, viruses have developed counter‐defense strategies such as anti‐restriction and anti‐CRISPR proteins, contributing to complex virus‐host interactions [7]. These antagonistic dynamics promote genetic variability and microdiversity (i.e., intra‐population genetic variation) [8]. Although these antagonistic dynamics have been extensively studied in shallow‐water environments [9], their specific presence in hadal trench sediments remain poorly understood. This is particularly the case with respect to how viral community structure, functional capacity and host association patterns vary across depth‐stratified sediment environments.

To address this gap, the adaptive survival strategies and interactions of host and viruses were analyzed, based on metagenomic analysis of the 28 samples from the Yap Trench and the Western Caroline Basin. The Yap Trench, which reaches a depth of 8527 mbs near the southeastern edge of the Philippine Sea Plate [1], because of its gentler slope, lower seismicity and decreased particle flow, provides a continuous depth gradient from shallow basins into the hadal zone [10]. In addition to patterns of viral diversity, viral anti‐defence protein between viruses and their prokaryotic hosts were also investigated. This study extends the understanding of the ecological patterns of viruses in sediments around trenches and elucidates how extreme hadal conditions shape virus‐host interactions and viral adaptation.

**Novel DNA viruses from sediments around the Yap Trench and Western Caroline Basin (YTWCB)**

In this study, 28 sediment metagenomes from 14 stations spanning multiple sediment depths were collected from the YTWCB in the western Pacific Ocean (Figure 1A). The environmental characteristics are shown in Table S1. Among the recovered viral sequences, 514 circular vOTUs and 316 high‐quality vOTUs were identified by CheckV (Figure 1B,C). To account for the hierarchical sampling design, community comparisons were conducted at the station level. Non‐metric multidimensional scaling (NMDS) analysis showed that the viral communities in YTWCB were separated into two groups across the carbonate compensation depth (CCD) boundary (stress = 0.05; PERMANOVA *p* = 0.005; *R* ^2^ = 0.151; Figure 1D). Samples below the CCD were designated as the CCD‐Below group, whereas those above the CCD were designated as the CCD‐Up group. This partition coincided with clear differences in sediment type, with the CCD‐Below group dominated by siliceous ooze and the CCD‐Up group dominated by calcareous ooze (Table S1). Multivariate dispersion did not differ significantly between groups (permutest of betadisper, *p* = 0.938), indicating that the separation primarily reflected differences in community composition rather than unequal within‐group dispersion. Viral Shannon‐Wiener diversity was higher in the CCD‐Below group, but the difference was not significant after multiple‐testing correction (Wilcoxon *p* = 0.043; BH‐FDR = 0.129; Figure 1E). According to geNomad annotation, most vOTUs (98.26%, *n* = 5466) were assigned to the order *Caudovirales*. Among the vOTUs classified at the family level, *Autographiviridae* (23.10%, *n* = 15), *Zobellviridae* (20.81%, *n* = 19), *Kyanoviridae* (15.35%, *n* = 12), and *Herelleviridae* (14.71%, *n* = 26) were the dominant families (Figure 1F, Table S2). Mantel analysis at the station level showed that viral community structure was mainly associated with latitude and seafloor depth (Figure 1G). Because calcium carbonate becomes undersaturated at abyssal to hadal depths, calcareous particles are progressively dissolved below the CCD, resulting in a transition from calcareous to siliceous ooze with increasing water depth [11]. This depth‐dependent shift in sediment type likely contributed to the observed differences in viral community structure between the two groups. The higher proportion of temperate viruses at hadal stations was treated as a descriptive pattern rather than formal enrichment evidence (Figure S1).

**Figure 1:**
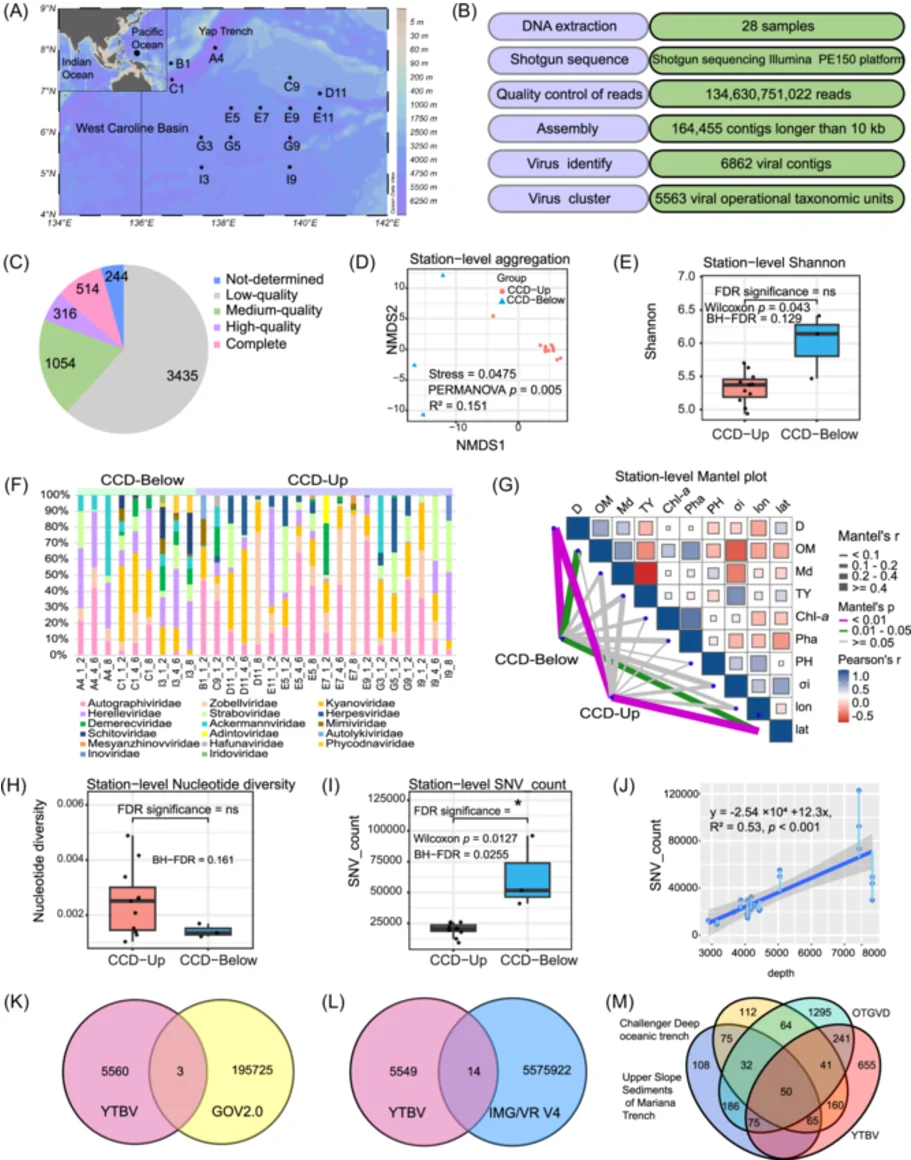
Overview of viruses in sediments from the Yap Trench and Western Caroline Basin (YTWCB). (A) Sediment sampling stations. (B) Overview of the experimental workflow and bioinformatic pipeline. (C) Quality distribution of recovered viral genomes. (D) Non‐metric multidimensional scaling (NMDS) analysis of viral community composition at the station level. Samples were grouped into CCD‐Below and CCD‐Up categories. Group separation was evaluated by PERMANOVA (*p* = 0.005, *R* ^2^ = 0.151), and multivariate dispersion did not differ significantly between groups (permutest of betadisper, *p* = 0.938). (E) Comparison of viral Shannon–Wiener diversity between the CCD‐Below and CCD‐Up groups at the station level (Wilcoxon *p* = 0.043; BH‐FDR = 0.129). (F) Family‐level taxonomic composition of sediment viruses. (G) Mantel test showing associations between viral community structure and environmental factors (D, seafloor water depth; Md, median grain size; YT, silt–clay content; Chl‐*a*, chlorophyll a content; Pha, pheophorbide; OM, organic matter content; σi, sorting coefficient; lon, longitude; lat, latitude; PH, Pondus Hydrogenii). (H) Comparison of nucleotide diversity (π) at the viral genome level between the CCD‐Below and CCD‐Up groups at the station level (Wilcoxon *p* = 0.161; BH‐FDR = 0.161). (I) Comparison of SNV counts at the viral genome level between the CCD‐Below and CCD‐Up groups at the station level (Wilcoxon *p* = 0.0127; BH‐FDR = 0.0255). (J) Linear regression analysis between viral SNV counts and seafloor water depth at the genome level. (K) Species‐level comparison of sediment viruses with the GOV 2.0 database based on 95% average nucleotide identity (ANI). (L) Species‐level comparison of sediment viruses with the IMG/VR V4 database based on 95% ANI. (M) Genus‐level viral clustering of sediment viruses with the Oceanic Trench Viral Genome Dataset (OTVGD), upper slope sediments of the Mariana Trench, and Challenger Deep trench viromes based on vConTACT2 results. Positive rank‐biserial values indicate higher values in the CCD‐Below group, whereas negative values indicate higher values in the CCD‐Up group.

To further examine differences between the two groups, viral microdiversity was assessed using nucleotide diversity (*π*) and single‐nucleotide variant (SNV) counts. Nucleotide diversity (*π*) did not differ significantly between groups (Wilcoxon *p* = 0.161; BH‐FDR = 0.161; Figure 1H), whereas SNV counts were significantly higher in the CCD‐Below group than in the CCD‐Up group (Wilcoxon *p* = 0.0127; BH‐FDR = 0.0255; Figure 1I). In addition, the number of SNVs was positively related to water depth (*p* < 0.001; Figure 1J), consistent with previous observations from the Mediterranean Sea, which also showed depth‐dependent trends in viral microdiversity [12]. These results indicate that increased SNV accumulation in CCD‐Below was the main microdiversity pattern associated with deeper sediments.

To assess the relationship between YTWCB viruses and currently available marine viral datasets, YTWCB vOTUs were compared with the Global Ocean Viromes (GOV 2.0) and IMG/VR v4 databases and were further clustered with hadal‐related viral datasets (Figure 1K and Table S3). At the species level, only three and four vOTUs shared >95% nucleotide similarity with sequences in GOV 2.0 and IMG/VR v4, respectively (Figure 1L), indicating limited overlap with currently available reference databases. At the genus level, however, vConTACT2 clustering identified a subset of viral clusters shared with other hadal trench datasets (Figure 1M), suggesting broader relatedness at higher taxonomic resolution. Because currently available hadal sediment virus remains limited and unevenly represented, these comparisons are intended primarily as descriptive contextualization rather than formal statistical evidence. Within this context, YTWCB viruses appear highly distinct at the species level while retaining detectable affiliations with other hadal viruses at the genus level.

**Trench viruses have a diverse range of ecological functions associated with deep‐sea adaptation**

To explore virus–host interactions, prokaryotic community structure was characterized from the same metagenomic datasets. A total of 427 metagenome‐assembled genomes (MAGs) were identified, including 390 bacterial and 37 archaeal genomes, with *Proteobacteria*, *Thermoproteota*, *Acidobacteriota*, *Methylomirabilota*, *Chloroflexota*, and *Planctomycetota* as the dominant phyla. Viral abundance broadly tracked host abundance, but clear depth‐related differences were observed (Figures 2A, S2, Table S4). Predicted associations with *Dehalococcoidia* were more frequently observed in the CCD‐Below group than in the CCD‐Up group. Because host assignments inferred from iPHoP, tRNA, and BLAST may involve possible one‐to‐many linkages, this pattern is interpreted here as a descriptive association rather than formal enrichment evidence. Given the important role of *Dehalococcoidia* in anaerobic carbon cycling through reductive dehalogenation of organohalides [13], this pattern may suggest a potential ecological link between viruses and carbon‐transforming processes in deeper sediments.

**Figure 2:**
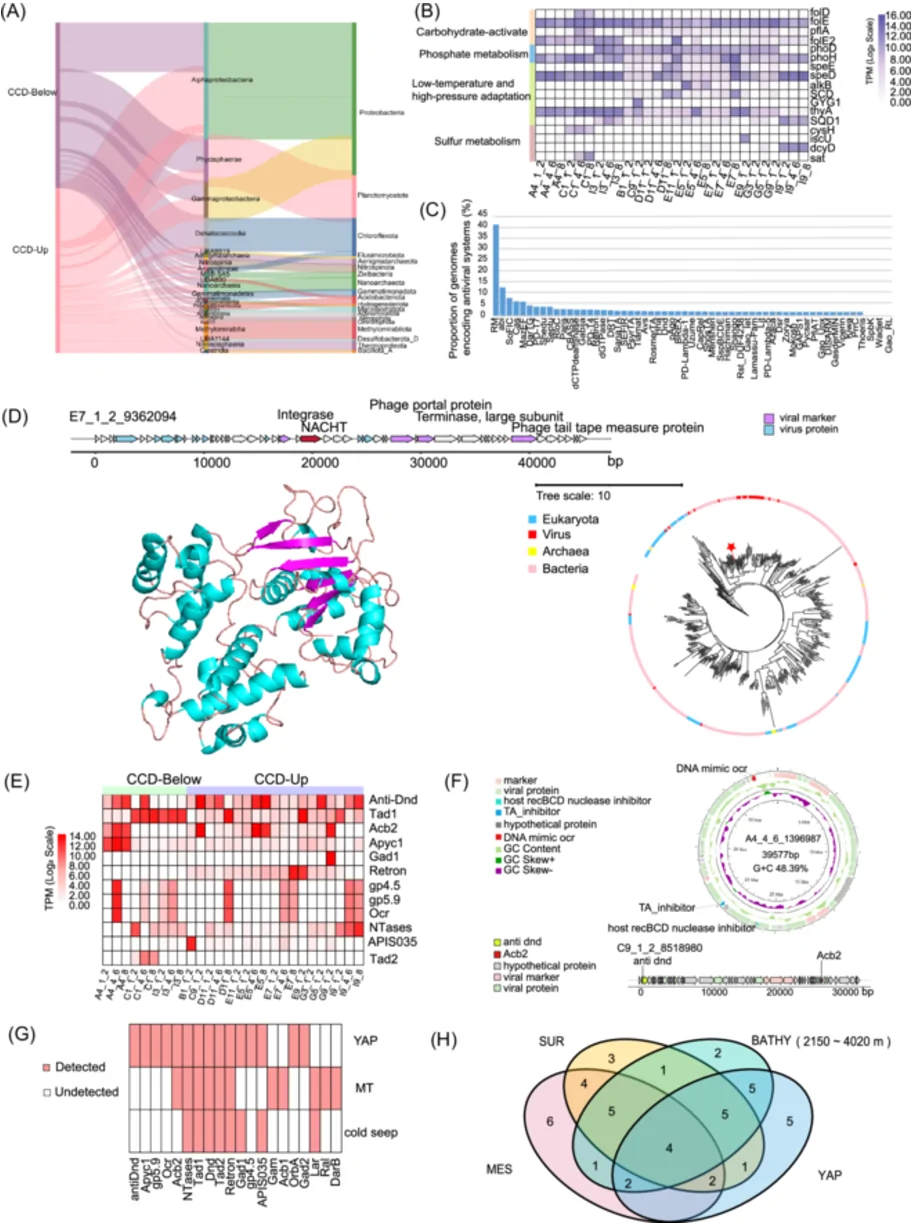
Virus–host interactions from the Yap Trench and Western Caroline Basin (YTWCB) sediments. (A) Virus–host linkages based on relative abundance patterns. (B) Relative abundance of virus‐encoded genes annotated against the KEGG database. (C) Diversity of antiviral defense systems identified in prokaryotic genomes. (D) Gene organization, predicted tertiary structures, and sequence‐based phylogenetic relationships of phage‐encoded NACHT proteins. The asterisk indicates the NACHT protein identified in this study. (E) Distribution of prokaryotic immune system suppressor proteins encoded by high‐quality viral genomes from YTWCB. (F) Representative gene maps of high‐quality viral genomes encoding multiple prokaryotic immune system suppressor proteins. (G) Distribution of prokaryotic immune system suppressor proteins encoded by high‐quality viral genomes from the Mariana Trench (MT), the Yap Trench (YAP), and cold seep sediments. (H) Distribution of prokaryotic immune system suppressor proteins encoded by high‐quality viral genomes in GOV 2.0 (BATHY, bathypelagic zone; MES, mesopelagic zone; SUR, surface zone) and this study.

To persist under these extreme conditions, viruses encode diverse functional and compositional adaptations. Among 255,794 predicted ORFs, genes of unknown function were the largest fraction. Annotated genes included abundant HNH endonucleases (*n* = 1079), DNA methylases (*n* = 311), and other DNA modification enzymes. A total of 1986 putative auxiliary viral genes (AVGs) were identified, including 399 CAZyme‐related proteins, mainly glycoside transferases (*n* = 321) and carbohydrate‐binding modules (*n* = 43), together with 35 proteins involved in lipid metabolism. In addition, AVGs were potentially associated with sulfur metabolism and adaptation to low‐temperature, high‐pressure conditions, pointing to metabolic capacities that may facilitate viral persistence in extreme trench sediments (Figure 2B). Viral protein stoichiometry also reflected environmental adaptation: higher carbon atoms per residue side chain (C‐ARSC) in groundwater viruses indicated utilization of carbon‐rich resources, whereas reduced nitrogen content was consistent with adaptation to nitrogen‐limited conditions.

These viral functions operate within a host environment shaped by substantial antiviral pressure. Host genomes encoded 1679 antiviral genes across 71 families, dominated by restriction‐modification (RM) systems (40.56%, *n* = 378), followed by abortive infection (Abi), SoFIC, and CRISPR‐Cas systems (Figure 2C). CRISPR‐Cas systems were detected in a subset of recovered MAGs, although their overall representation was lower than that of RM and Abi systems in this dataset; detailed CRISPR‐related results are provided in the Table S5. Temperate phages also carried RosmerTA, NixI, and NACHT, and viral NACHT proteins clustered with bacterial homologs, consistent with possible horizontal gene transfer (Figure 2D). In parallel, viruses encoded multiple counter‐defense systems. DNA methylases may protect viral genomes from RM‐mediated cleavage, and 34 immune‐suppressing proteins were detected, with Anti‐Dnd, *Tad1*, and *Acb2* being the most prevalent (Figures 2E,F). Comparison with available datasets from the Guaymas Basin, Mariana Trench, and deep‐sea cold seep sediments showed that several defense/counter‐defense categories were shared across deep‐sea environments, whereas others were found only in YTWCB among the datasets examined (Figures 2G, 2H, S3‐S7). However, these comparisons should be interpreted cautiously because the datasets differ in sample number, sequencing depth, assembly quality, viral recovery, and annotation completeness. Therefore, these patterns are considered descriptive differences in representation rather than formal evidence of enrichment.

Microdiversity analyses further indicated pervasive purifying selection across viral genes (*n* = 54,482; pN/pS < 1), with 40,222 genes under particularly strong constraint (pN/pS < 0.3). These conserved genes were mainly structural proteins, whereas receptor‐binding proteins accumulated more nonsynonymous substitutions, consistent with increased sequence diversification in host‐recognition functions. The enrichment of both strongly constrained genes and genes with greater sequence diversification in the CCD‐Below group relative to the CCD‐Up group suggests a more heterogeneous selective regime in deeper sediments. Together, these patterns support two complementary adaptive strategies: conservation of core viral functions and diversification of host‐interaction genes.

## AUTHOR CONTRIBUTIONS

**Yantao Liang** and **Min Wang**: Supervision, conceptualization, funding acquisition, and project administration. **Yue Su**: Methodology, writing, and original draft. **Hao Yu** and **Shujuan Sun**: Data curation and software. **Kaiyang Zheng**, **Ziyue Wang**, **Chen Gao**, and **David Paez‐Espino**: Data curation and visualization. **Hongbing Shao** and **Andrew McMinn**: Writing, review, and editing. **Guangxue Li** and **Xiaoshou Liu**: Resources and environmental factors. All authors contributed to the article and approved the submitted version.

## CONFLICT OF INTEREST STATEMENT

The authors declare no conflicts of interest.

## ETHICS STATEMENT

No animals or humans were involved in this study.

## Supporting information


**Figure S1:** Percentage abundance of lytic and temperate viruses in the sediment viruses of the Yap Trench and West Caroline Basin.
**Figure S2:** Bubble map of the relative abundance of prokaryotes at the phylum level in Yap Trench and West Caroline Basin.
**Figure S3:** The prokaryotic immune system suppressor proteins of the virus.
**Figure S4:** Maximum‐likelihood phylogeny of Tad2 proteins. Tad2 proteins from this study and reference deep‐sea datasets were used to reconstruct a maximum‐likelihood phylogenetic tree. Sequences from this study, cold seep, and MT (the Mariana Trench) are shown in purple, blue, and beige, respectively. Circles indicate bootstrap support values of 70–100. Scale bar, amino acid substitutions per site.
**Figure S5:** Maximum‐likelihood phylogeny of Tad1 proteins. Tad1 proteins from this study and reference deep‐sea datasets were used to reconstruct a maximum‐likelihood phylogenetic tree. Sequences from this study, cold seep, and MT (the Mariana Trench) are shown in purple, blue, and beige, respectively. Circles indicate bootstrap support values of 70–100. Scale bar, amino acid substitutions per site.
**Figure S6:** Maximum‐likelihood phylogeny of NTases (nucleotidyltransferases) proteins. NTases proteins from this study and reference deep‐sea datasets were used to reconstruct a maximum‐likelihood phylogenetic tree. Sequences from this study, cold seep, and MT (the Mariana Trench) are shown in purple, blue, and beige, respectively. Circles indicate bootstrap support values of 70–100. Scale bar, amino acid substitutions per site.
**Figure S7:** Maximum‐likelihood phylogeny of Anti‐Dnd proteins. Anti‐Dnd proteins from this study and reference deep‐sea datasets were used to reconstruct a maximum‐likelihood phylogenetic tree. Sequences from this study, cold seep, MT (the Mariana Trench), and YT (the YAP Trench) are shown in purple, blue, beige, and pink, respectively. Circles indicate bootstrap support values of 70–100. Scale bar, amino acid substitutions per site.
**Table S1:** Basic information about YAP trench sediment.
**Table S2:** Taxonomy of viruses in sediments from Yap Trench and West Caroline Basin in the Western Pacific Ocean based on the geNomad.
**Table S3:** The clustering information of sediment viruses in Yap Trench and West Carolina Basin with IMG/VR v4 database, GOV2.0 database, OTGVD database, upper slope sediments of the Mariana Trench viromes, Challenger Deep oceanic trench viromes and North of YAP Trench virus.
**Table S4:** Putative virus‐host linkages.
**Table S5:** The spacer of MAGs.

## Data Availability

The data that support the findings of this study are openly available in CRA025041 at https://share.cncb.ac.cn/M4MNO8VL. The clean reads data reported in this paper have been deposited in the Genome Sequence Archive in the National Genomics Data Center [14], China National Center for Bioinformation/Beijing Institute of Genomics, Chinese Academy of Sciences (GSA: CRA025041; https://share.cncb.ac.cn/M4MNO8VL). The data and scripts used are saved in GitHub (https://github.com/yuesu602/YTWCB_result/tree/main/YTWCB_final_public_repo/YTWCB). Supplementary materials (methods, figures, tables, scripts, graphical abstract, slides, videos, Chinese translated version and update materials) may be found in the online DOI or iMeta Science http://www.imeta.science/imetaomics/.
