# Supplementary material for: Viruses in the sediments around the trenches harbor diverse survival mechanisms
Source: IMetaOmics. 2026 Jul 26;3(3):e70124. doi: 10.1002/imo2.70124 (PMC13674397; doi:10.1002/imo2.70124)
Supplement: Supplementary file 1 [file IMO2-3-e70124-s001.docx]

Supporting information to

**Viruses in the sediments around the trenches harbor diverse survival mechanisms.**

**Running title:** Survival Mechanisms of Trench Viruses

Yue Su^1^, Hao Yu^1^, Shujuan Sun^1^, Kaiyang Zheng^1^, Ziyue Wang^1^, Chen Gao^1,2^, Guangxue Li^3^, Xiaoshou Liu^1^, Hongbing Shao^1,4^, David Paez-Espino^5^, Andrew McMinn^1,6^, Yantao Liang^1,4*^, Min Wang^1,2,4*^

^1^ MoE Laboratory of Evolution and Marine Biodiversity, College of Marine Life Sciences, Institute of Evolution and Marine Biodiversity, Frontiers Science Center for Deep Ocean, Center for Ocean Carbon Neutrality, Ocean University of China, Qingdao, China.

^2^ Haide College, Ocean University of China, Qingdao, China.

^3^ College of Marine Geosciences, Ocean University of China

^4^ UMT-OUC Joint Academic Centre for Marine Studies, Qingdao, China.

^5^ Ancilia Biosciences, Inc., Alexandria Launch labs, New York, NY, USA.

^6^ Institute for Marine and Antarctic Studies, University of Tasmania, Hobart, TAS, Australia.

^*^ Correspondence: [liangyantao@ouc.edu.cn](mailto:liangyantao@ouc.edu.cn) (Y.L.); [mingwang@ouc.edu.cn](mailto:mingwang@ouc.edu.cn) (M.W.)

**METHODS**

**Sample collection**

Samples were collected from 14 stations near the Yap Trench aboard R/V Hai Da (Figure 1a) from May to July 2017. Stations A4 and C1 are located in the southern Yap Trench (water depths > 7000 m), station B1 is located in the back-arc basin (Yap Island Arc), and the remaining stations are located in the Western Caroline Basin. A Gray-O'Hara box sampler measuring 0.25 m^2^ was used to collect the sediment cores [1], and the top 10 cm of sediment was divided into three depths: 1 to 2 cm, 4 to 6 cm, and 8 cm. The 28 sediment samples were selected from these, seven (A4, C1, D11, E5, E7, I3, and I9) from the surface layer at depths of 1 ~ 2 cm, 4 ~ 6 cm, and 8 cm, and other seven (B1, C9, E9, E11, G3, G5, and G9) from 1 ~ 2 cm of sediment. The samples were refrigerated at -80°C until DNA extraction. The physical and chemical characteristics of the sediments are presented in Supplementary Table S1.

**The DNA extraction and metagenomic sequencing**

The sediment samples were shipped to Novogene Biotechnology in Beijing, where a Magnetic Soil and Stool DNA Kit was used following the manufacturer's instructions to extract nucleic acid. The high-throughput sequencing platform NovaSeq 6000 from Illumina was used to sequence the extracted whole DNA (double-end sequencing, 2 × 150 bp).

**Quality control of reads, assembly, and virus identification**

To improve the confidence of the data, clean data was performed using fastp to remove adapters and sequences containing indeterminate bases [2]. Subsequently, a Perl script was applied to filter reads based on Phred Score (Q-value) by: (1) discarding sequences if > 20% of bases had a Q-value < 20, and (2) discarding sequences if > 30% of bases had a Q-value < 30 [3]. Filtered reads were assembled using the default parameters of the MEGAHIT [4] software, and contigs < 500 bp were removed using seqkit [5].

Viral sequences ≥ 1.5 kb were identified using VirSorter, VirFinder, CAT [6], and geNomad [7]. First screened as candidate viral sequences if they were classified into VirSorter categories 1–6 and/or had a VirFinder score ≥ 0.7 with *p* < 0.05 [8,9]. Candidate contigs were classified as viral if they met one of the following criteria: (i) VirFinder score ≥ 0.9 with *p* < 0.05; (ii) VirSorter category 1 or 2; (iii) concurrent support from VirSorter (categories 1 – 6) and VirFinder (score ≥ 0.7 with *p* < 0.05); or (iv) CAT assignment to viral or unclassified domains, with < 40% of the contig classified as bacterial, archaeal, or eukaryotic [10]. Identified viral sequences were processed with CheckV [11] to remove host-derived cellular boundaries, and the resulting boundary-corrected contigs were further evaluated with geNomad. Only sequences with a geNomad score ≥ 0.9 and at least one viral hallmark gene were retained. Viral contigs ≥ 10 kb, or ≥ 1.5 kb if circular, were used for downstream analyses. The CD-HIT [12] v4.8.1 (parameters:-c 0.95 -M 0 -aS 0.8 -T 8 -n 9 -G 0) was used for 95% Average Nucleotide Identity (ANI) clustering to generate viral Operational Taxonomic Units (vOTUs). Virus sequences were annotated with geNomad (v1.7.7) [13]. BACPHLIP (minimum score ≥ 0.8) [14] and VIBRANT [15] were used to predict the viral lifestyle. When VIBRANT and BACPHLIP predictions were conflicting, the information from the genome annotation was prioritized. Lysogeny-specific genes (i.e., integrase, recombinase, transposase, excisionase, CI/Cro repressor, and parAB) were manually detected.

The inStrain was run (-average read depth ≥ 10×, genome coverage breadth ≥ 50%) to calculate Nucleotide diversity (π), single nucleotide variant (Single nucleotide variants (SNVs), and the ratio of non-synonymous to synonymous polymorphisms (pN/pS) [16].

**Comparison with other environmental viruses**

To investigate the specificity of the viruses, the viruses were clustered with lengths greater than 10 kbp or circular in the Global Ocean DNA virome (GOV 2.0) database [11]. The main principle is to use nucleic acid sequences for library construction, and then clustering based on 95% Average Nucleotide Identity (ANI), and 85% aligned fraction ratio (AF) [11]. In addition, 5,575,936 complete and high-quality viral sequences from the IMG/VR v4 [13] database, viral sequences from the oceanic trench viral genome dataset (OTVGD) [17], Challenger Deep [18], and upper slope of the Mariana Trench [19], were downloaded and clustered.

**Analysis of virus-host interactions**

The contigs were binned (parameters: –maxbin2 –metabat2 –concoct) and refined (parameters: -c 70 -x 5) using the metaWRAP (v 1.3.2) [20]. Using dRep v3.2.0 (parameters: -comp 70 -con 5 -sa 0.95), the 427 species-level metagenome-assembled genomes (MAGs) were acquired with 95% average nucleotide identity (ANI) aggregation and de-redundancy of all resulting bin sets. The taxonomy of MAGs was assigned by GTDB-Tk (GTDB r226) [21]. Prodigal software was utilized for the prediction of Open reading frames (ORFs) of MAGs [22]. The functional annotation was using an online website (GhostKOALA (kegg.jp)) based on the Kyoto Encyclopedia of Genes and Genomes (KEGG) database [23].

The potential prediction of hosts is based on a combination of the iPHoP [24] and ARAGORN [25]. The ARAGORN software was used to identify tRNA sequences in the vOTUs and MAGs sequences. All identified tRNA sequences were subjected to a bi-directional blastn comparison, and only results with more than 90% identity and coverage on both sides of the forward and backward comparison of tRNA sequences from the vOTUs and the MAGs were considered to indicate the viral host relationship. Host-virus network diagrams and Sankey diagrams were visualized by Gephi [26] and online sites (https://sankeymatic.com/build/). DefenseFinder software was used to identify potential antiviral systems in host genomes and high-quality viral genomes [27].

**Functional analysis of viral genes**

Prodigal software was utilized for the prediction of Open reading frames (ORFs) of vOTUs [22]. All ORFs were compared with the Conserved Domain Database (CDD) by RPSBLAST [28] and Pfam databases (e-value < 1e-5, score > 40). The Evolutionary Genealogy of Genes: Non-supervised Orthologous Groups (eggNOG) database was performed using eggNOG-mapper 2.1.12 to predict the functions of proteins [29].

To identify virus-encoded auxiliary metabolic genes, all vOTUs sequences were entered into VirSorter 2 (prep-for-dramv mode) [30], and the results were imported into DRAM-v [31]. Genes with auxiliary scores of 1 ~ 3 and labeled M and F were considered potential AVGs. The potential AVGs were also annotated based on the KEGG database and VIBRANT [23]. The carbohydrate-active enzymes (CAZymes) based on Hidden Markov Models (HMMs) of the CAZyme family were used by the dbCAN software [32].

Reference sequences of bacterial defense-system inhibitory proteins were compiled from the literature and used to identify homologous inhibitory proteins in the viral protein(e-value < 1 × 10^-5^) [33,34]. Because such genes may be affected by host contamination, especially in temperate or proviral sequences, candidate defense- and counter-defense-associated genes were manually inspected in high-quality temperate genomes based on genomic context, host–virus boundaries, and the presence of neighboring viral hallmark or viral-like genes. Viral hallmark or viral-like genes were defined primarily based on annotations associated with virion structure, genome packaging, and phage lysis modules, including capsid, terminase, portal, baseplate, prohead, tape measure, tail, neck, virion, and holin proteins. Replication- and recombination-related proteins, such as helicases, primases, endonucleases, exonucleases, resolvases, and SSB proteins, were considered only as supportive evidence rather than as standalone viral markers [35].

Protein modeling and prediction were performed using the Phyre2 online website [36], and visualization of 3D structures was performed using PyMOL [37]. The abundance of each virally encoded AVG was inferred from the abundance of the viral genome carrying that AVG, rather than from reads mapped directly to individual AVG loci, in order to minimize potential bias from host-derived reads. The functional gene heatmaps were produced using TBtools [38]. The genome annotation visualization using Proksee online [39].

**Statistical analysis**

The relative abundance of viruses and hosts was calculated using CoverM (--min-read-percent-identity 0.95 --min-read-aligned-percent 0.75) (GitHub, wwood/CoverM: Read coverage calculator for metagenomics). All statistical analyses were performed in R version 4.2.3 using the R packages Vegan and ape to estimate the α-diversity (the Chao1 index, Simpson index, and Shannon-Wiener index). The Bray-Curtis similarity was generated from the OTUs table of viral abundance (TPM) using the vegdist function. The tests were examined using the Wilcox test and visualized using ggplot2. The Mantel Test for Correlation between the OTUs and the environmental factors was calculated using the Vegan package; the correlation matrix was heatmapped using the ggcor package (https://github.com/hannet91/ggcor); and the visualizations using the ggplot2 package. Linear regression analysis using tidyverse (https://github.com/tidyverse/tidyverse) and ggpmisc (https://github.com/aphalo/ ggpmisc) packages.

**References**

1. Wang, Xiaoxiao, Xiaoshou Liu, and Jishang Xu. 2019. “Distribution Patterns of Meiofauna Assemblages and Their Relationship With Environmental Factors of Deep Sea Adjacent to the Yap Trench, Western Pacific Ocean.” *Frontiers in Marine Science* 6: 735. https://doi.org/10.3389/fmars.2019.00735

2. Chen, Shifu, Yanqing Zhou, Yaru Chen, and Jia Gu. 2018. “fastp: an ultra-fast all-in-one FASTQ preprocessor.” *Bioinformatics* 34: i884–i890. https://doi.org/10.1093/bioinformatics/bty560

3. Gao, Chen, Yantao Liang, Yong Jiang, David Paez-Espino, Meiaoxue Han, Chengxiang Gu, et al. 2022. “Virioplankton assemblages from challenger deep, the deepest place in the oceans.” *iScience* 25: 104680. https://doi.org/10.1016/j.isci.2022.104680

4. Li, Dinghua, Ruibang Luo, Chi-Man Liu, Chi-Ming Leung, Hing-Fung Ting, Kunihiko Sadakane, et al. 2016. “MEGAHIT v1.0: A fast and scalable metagenome assembler driven by advanced methodologies and community practices.” *Methods* 102: 3–11. https://doi.org/10.1016/j.ymeth.2016.02.020

5. Shen, Wei, Shuai Le, Yan Li, and Fuquan Hu. 2016. “SeqKit: A Cross-Platform and Ultrafast Toolkit for FASTA/Q File Manipulation.” *PLOS ONE* 11: e0163962. https://doi.org/10.1371/journal.pone.0163962

6. Gregory, Ann C., Ahmed A. Zayed, Nádia Conceição-Neto, Ben Temperton, Ben Bolduc, Adriana Alberti, et al. 2019. “Marine DNA Viral Macro- and Microdiversity from Pole to Pole.” *Cell* 177: 1109-1123.e14. https://doi.org/10.1016/j.cell.2019.03.040

7. Camargo, Antonio Pedro, Simon Roux, Frederik Schulz, Michal Babinski, Yan Xu, Bin Hu, et al. 2024. “Identification of mobile genetic elements with geNomad.” *Nature Biotechnology* 42: 1303–1312. https://doi.org/10.1038/s41587-023-01953-y

8. Ren, Jie, Nathan A. Ahlgren, Yang Young Lu, Jed A. Fuhrman, and Fengzhu Sun. 2017. “VirFinder: a novel k-mer based tool for identifying viral sequences from assembled metagenomic data.” *Microbiome* 5: 69. https://doi.org/10.1186/s40168-017-0283-5

9. Roux, Simon, Francois Enault, Bonnie L. Hurwitz, and Matthew B. Sullivan. 2015. “VirSorter: mining viral signal from microbial genomic data.” *PeerJ* 3: e985. https://doi.org/10.7717/peerj.985

10. von Meijenfeldt, F. A. Bastiaan, Ksenia Arkhipova, Diego D. Cambuy, Felipe H. Coutinho, and Bas E. Dutilh. 2019. “Robust taxonomic classification of uncharted microbial sequences and bins with CAT and BAT.” *Genome Biology* 20: 217. https://doi.org/10.1186/s13059-019-1817-x

11. Nayfach, Stephen, Antonio Pedro Camargo, Frederik Schulz, Emiley Eloe-Fadrosh, Simon Roux, and Nikos C. Kyrpides. 2021. “CheckV assesses the quality and completeness of metagenome-assembled viral genomes.” *Nature Biotechnology* 39: 578–585. https://doi.org/10.1038/s41587-020-00774-7

12. Fu, Limin, Beifang Niu, Zhengwei Zhu, Sitao Wu, and Weizhong Li. 2012. “CD-HIT: accelerated for clustering the next-generation sequencing data.” *Bioinformatics* 28: 3150–3152. https://doi.org/10.1093/bioinformatics/bts565

13. Camargo, Antonio Pedro, Stephen Nayfach, I-Min A Chen, Krishnaveni Palaniappan, Anna Ratner, Ken Chu, et al. 2023. “IMG/VR v4: an expanded database of uncultivated virus genomes within a framework of extensive functional, taxonomic, and ecological metadata.” *Nucleic Acids Research* 51: D733–D743. https://doi.org/10.1093/nar/gkac1037

14. Hockenberry, Adam J., and Claus O. Wilke. 2021. “BACPHLIP: predicting bacteriophage lifestyle from conserved protein domains.” *PeerJ* 9: e11396. https://doi.org/10.7717/peerj.11396

15. Kieft, Kristopher, Zhichao Zhou, and Karthik Anantharaman. 2020. “VIBRANT: automated recovery, annotation and curation of microbial viruses, and evaluation of viral community function from genomic sequences.” *Microbiome* 8: 90. https://doi.org/10.1186/s40168-020-00867-0

16. Olm, Matthew R., Alexander Crits-Christoph, Keith Bouma-Gregson, Brian A. Firek, Michael J. Morowitz, and Jillian F. Banfield. 2021. “inStrain profiles population microdiversity from metagenomic data and sensitively detects shared microbial strains.” *Nature Biotechnology* 39: 727–736. https://doi.org/10.1038/s41587-020-00797-0

17. Jian, Huahua, Yi Yi, Jiahua Wang, Yali Hao, Mujie Zhang, Siyuan Wang, et al. 2021. “Diversity and distribution of viruses inhabiting the deepest ocean on Earth.” *The ISME Journal* 15: 3094–3110. https://doi.org/10.1038/s41396-021-00994-y

18. Zhou, Ying-Li, Paraskevi Mara, Dean Vik, Virginia P. Edgcomb, Matthew B. Sullivan, and Yong Wang. 2022. “Ecogenomics reveals viral communities across the Challenger Deep oceanic trench.” *Communications Biology* 5: 1055. https://doi.org/10.1038/s42003-022-04027-y

19. Zhao, Jiulong, Hongmei Jing, Zengmeng Wang, Long Wang, Huahua Jian, Rui Zhang, et al. 2022. “Novel Viral Communities Potentially Assisting in Carbon, Nitrogen, and Sulfur Metabolism in the Upper Slope Sediments of Mariana Trench.” *mSystems* 7: e01358-21. https://doi.org/10.1128/msystems.01358-21

20. Uritskiy, Gherman V., Jocelyne DiRuggiero, and James Taylor. 2018. “MetaWRAP—a flexible pipeline for genome-resolved metagenomic data analysis.” *Microbiome* 6: 158. https://doi.org/10.1186/s40168-018-0541-1

21. Chaumeil, Pierre-Alain, Aaron J Mussig, Philip Hugenholtz, and Donovan H Parks. 2019. “GTDB-Tk: a toolkit to classify genomes with the Genome Taxonomy Database.” Bioinformatics 36(6): 1925–1927. https://doi.org/10.1093/bioinformatics/btz848

22. Hyatt, Doug, Gwo-Liang Chen, Philip F LoCascio, Miriam L Land, Frank W Larimer, and Loren J Hauser. 2010. “Prodigal: prokaryotic gene recognition and translation initiation site identification.” *BMC Bioinformatics* 11: 119. https://doi.org/10.1186/1471-2105-11-119

23. Kanehisa, Minoru, Yoko Sato, and Kanae Morishima. 2016. “BlastKOALA and GhostKOALA: KEGG Tools for Functional Characterization of Genome and Metagenome Sequences.” *Journal of Molecular Biology* 428: 726–731. https://doi.org/10.1016/j.jmb.2015.11.006

24. Roux, Simon, Antonio Pedro Camargo, Felipe H. Coutinho, Shareef M. Dabdoub, Bas E. Dutilh, Stephen Nayfach, et al. 2023. “iPHoP: An integrated machine learning framework to maximize host prediction for metagenome-derived viruses of archaea and bacteria.” *PLOS Biology* 21: e3002083. https://doi.org/10.1371/journal.pbio.3002083

25. Laslett, D. 2004. “ARAGORN, a program to detect tRNA genes and tmRNA genes in nucleotide sequences.” *Nucleic Acids Research* 32: 11–16. https://doi.org/10.1093/nar/gkh152

26. Bastian, Mathieu, Sebastien Heymann, and Mathieu Jacomy. 2009. “Gephi: An Open Source Software for Exploring and Manipulating Networks.” *Proceedings of the International AAAI Conference on Web and Social Media* 3: 361–362. https://doi.org/10.1609/icwsm.v3i1.13937

27. Tesson, Florian, Alexandre Hervé, Ernest Mordret, Marie Touchon, Camille d’Humières, Jean Cury, et al. 2022. “Systematic and quantitative view of the antiviral arsenal of prokaryotes.” *Nature Communications* 13: 2561. https://doi.org/10.1038/s41467-022-30269-9

28. Lu, Shennan, Jiyao Wang, Farideh Chitsaz, Myra K Derbyshire, Renata C Geer, Noreen R Gonzales, et al. 2020. “CDD/SPARCLE: the conserved domain database in 2020.” *Nucleic Acids Research* 48: D265–D268. https://doi.org/10.1093/nar/gkz991

29. Hernández-Plaza, Ana, Damian Szklarczyk, Jorge Botas, Carlos P Cantalapiedra, Joaquín Giner-Lamia, Daniel R Mende, et al. 2023. “eggNOG 6.0: enabling comparative genomics across 12 535 organisms.” *Nucleic Acids Research* 51: D389–D394. https://doi.org/10.1093/nar/gkac1022

30. Guo, Jiarong, Ben Bolduc, Ahmed A. Zayed, Arvind Varsani, Guillermo Dominguez-Huerta, Tom O. Delmont, et al. 2021. “VirSorter2: a multi-classifier, expert-guided approach to detect diverse DNA and RNA viruses.” *Microbiome* 9: 37. https://doi.org/10.1186/s40168-020-00990-y

31. Shaffer, Michael, Mikayla A Borton, Bridget B McGivern, Ahmed A Zayed, Sabina Leanti La Rosa, Lindsey M Solden, et al. 2020. “DRAM for distilling microbial metabolism to automate the curation of microbiome function.” *Nucleic Acids Research* 48: 8883–8900. https://doi.org/10.1093/nar/gkaa621

32. Yin, Yanbin, Xizeng Mao, Jincai Yang, Xin Chen, Fenglou Mao, and Ying Xu. 2012. “dbCAN: a web resource for automated carbohydrate-active enzyme annotation.” *Nucleic Acids Research* 40: W445–W451. https://doi.org/10.1093/nar/gks479

33. Yan, Yuchen, Jinfang Zheng, Xinpeng Zhang, and Yanbin Yin. 2024. “dbAPIS: a database of anti-prokaryotic immune system genes.” *Nucleic Acids Research* 52: D419–D425. https://doi.org/10.1093/nar/gkad932

34. Mayo-Muñoz, David, Rafael Pinilla-Redondo, Sarah Camara-Wilpert, Nils Birkholz, and Peter C. Fineran. 2024. “Inhibitors of bacterial immune systems: discovery, mechanisms and applications.” *Nature Reviews Genetics* 25: 237–254. https://doi.org/10.1038/s41576-023-00676-9

35. Zhu, Shuang, Zhijie Tan, Ziyu Guo, Huijian Zheng, Baoshan Zhang, Zhi Qin, et al. 2024. “Symbiotic virus-bacteria interactions in biological treatment of coking wastewater manipulating bacterial physiological activities.” *Water Research* 257: 121741. https://doi.org/10.1016/j.watres.2024.121741

36. Kelley, Lawrence A, Stefans Mezulis, Christopher M Yates, Mark N Wass, and Michael J E Sternberg. 2015. “The Phyre2 web portal for protein modeling, prediction and analysis.” *Nature Protocols* 10: 845–858. https://doi.org/10.1038/nprot.2015.053

37. Schiffrin, Bob, Sheena E. Radford, David J. Brockwell, and Antonio N. Calabrese. 2020. “PyXlinkViewer: A flexible tool for visualization of protein chemical crosslinking data within the PyMOL molecular graphics system.” *Protein Science* 29: 1851–1857. https://doi.org/10.1002/pro.3902

38. Chen, Chengjie, Hao Chen, Yi Zhang, Hannah R. Thomas, Margaret H. Frank, Yehua He, et al. 2020. “TBtools: An Integrative Toolkit Developed for Interactive Analyses of Big Biological Data.” *Molecular Plant* 13: 1194–1202. https://doi.org/10.1016/j.molp.2020.06.009

39. Grant, Jason R, Eric Enns, Eric Marinier, Arnab Mandal, Emily K Herman, Chih-yu Chen, et al. 2023. “Proksee: in-depth characterization and visualization of bacterial genomes.” *Nucleic Acids Research* 51: W484–W492. https://doi.org/10.1093/nar/gkad326

**Figure S1. Percentage abundance of lytic and temperate viruses in the sediment viruses of the Yap Trench and Western Caroline Basin.** Samples were divided into two groups based on the carbonate compensation depth (CCD): the CCD-Below group (below the CCD) and the CCD-Up group (above the CCD).

**Figure S2. Bubble map of the relative abundance of prokaryotes at the phylum level in Yap Trench and Western Caroline Basin.** Samples were divided into two groups based on the carbonate compensation depth (CCD): the CCD-Below group (below the CCD) and the CCD-Up group (above the CCD).

**Figure S3. The prokaryotic immune system suppressor proteins of the virus.** Comparison of the number of type prokaryotic immune system suppressor proteins encoded in the high-quality viral genome in different environments (YAP, this study; MT, the Mariana Trench sediment; and Cold seep sediment). The upset the number of type prokaryotic immune system suppressor proteins encoded in the high-quality viral genome.

**Figure S4. Maximum-likelihood phylogeny of Tad2 proteins. Tad2 proteins from this study and reference deep-sea datasets were used to reconstruct a maximum-likelihood phylogenetic tree.** Sequences from this study, cold seep, and MT (the Mariana Trench) are shown in purple, blue, and beige, respectively. Circles indicate bootstrap support values of 70–100. Scale bar, amino acid substitutions per site.

**Figure S5. Maximum-likelihood phylogeny of Tad1 proteins. Tad1 proteins from this study and reference deep-sea datasets were used to reconstruct a maximum-likelihood phylogenetic tree.** Sequences from this study, cold seep, and MT (the Mariana Trench) are shown in purple, blue, and beige, respectively. Circles indicate bootstrap support values of 70–100. Scale bar, amino acid substitutions per site.

**Figure S6. Maximum-likelihood phylogeny of NTases (nucleotidyltransferases) proteins. NTases proteins from this study and reference deep-sea datasets were used to reconstruct a maximum-likelihood phylogenetic tree.** Sequences from this study, cold seep, and MT (the Mariana Trench) are shown in purple, blue, and beige, respectively. Circles indicate bootstrap support values of 70–100. Scale bar, amino acid substitutions per site.

**Figure S7. Maximum-likelihood phylogeny of Anti-Dnd proteins.** Anti-Dnd proteins from this study and reference deep-sea datasets were used to reconstruct a maximum-likelihood phylogenetic tree. Sequences from this study, cold seep, MT (the Mariana Trench), and YT (the YAP Trench) are shown in purple, blue, beige, and pink, respectively. Circles indicate bootstrap support values of 70–100. Scale bar, amino acid substitutions per site.
